# Chemical Composition and Source Apportionment of Wintertime Airborne PM_2.5_ in Changchun, Northeastern China

**DOI:** 10.3390/ijerph18084354

**Published:** 2021-04-20

**Authors:** Shichun Zhang, Daniel Q. Tong, Mo Dan, Xiaobing Pang, Weiwei Chen, Xuelei Zhang, Hongmei Zhao, Yiyong Wang, Bingnan Shang

**Affiliations:** 1 Key Laboratory of Wetland Ecology and Environment, Northeast Institute of Geography and Agroecology, Chinese Academy of Sciences, 4888 Shengbei Street, Changchun 130102, China; chenweiwei@iga.ac.cn (W.C.); zhangxuelei@iga.ac.cn (X.Z.); zhaohongmei@iga.ac.cn (H.Z.); 2 Center for Spatial Information Science and Systems, George Mason University, Fairfax, VA 22030, USA; qtong@gmu.edu; 3 Beijing Municipal Institute of Labor Protection, Beijing 100054, China; danmo2001@126.com; 4 Institute of Environment, Zhejiang University of Technology, Hangzhou 310006, China; pangxbyuanj@163.com; 5 School of Geography and Environment, Baoji University of Arts and Sciences, Baoji 721013, China; wangyiyong@iga.ac.cn; 6 Department of Library, Jilin University of Finance and Economics, Changchun 130117, China; sbn305@163.com

**Keywords:** aerosol, PM_2.5_, measurement, Northeast China, source apportionment

## Abstract

This study presents field observations and laboratory analyses of wintertime airborne particulate matter (PM_2.5_) and its chemical components in the Changchun metropolitan area, the geographical center of northeastern China. Twenty-four hour PM_2.5_ filter samples were collected from 23 December 2011 to 31 January 2012 at four sites in the types of traffic, residential, campus, and a near-city rural village, respectively. Daily PM_2.5_ concentrations ranged from 49 to 466 µg m^−3^, with an arithmetic average of 143 µg m^−3^. Laboratory analyses showed that among all measured chemical species, mineral dust contributed the largest proportion (20.7%) to the total PM_2.5_ mass, followed by secondary inorganic aerosols (SIA, including SO_4_^2−^, NO_3_^−^ and NH_4_^+^), which constituted 18.8% of PM_2.5_ mass. Another notable feature of PM_2.5_ chemical composition was high halogen (Cl^−^ and F^−^) loadings at all sites, which was likely due to emissions from coal combustion, plastic manufacturing, and glass melting. Among the four sampling sites, the suburban site exhibited the highest PM_2.5_ levels and extremely high Cl^−^ and F^−^ loadings due to residential wood burning and nearby industrial facilities lacking effective emission controls. Our results report one of the earliest observations of PM_2.5_ composition in this region, providing a baseline of aerosol profiles of aerosol before PM_2.5_ was routinely measured by environmental protection agencies in China, which could be useful for assessing long-term trends of air quality and effectiveness of mitigation measures.

## 1. Introduction

Exposure to outdoor air pollution causes 4.2 million premature deaths worldwide every year, making air pollution the single largest environmental risk today [1]. Among all pollutants, ozone (O_3_) and PM_2.5_ (particulate matter with diameter <2.5 μm^−3^) are most closely associated with increased occurrence of lung cancer and cardiovascular diseases [2,3]. A large portion of the global health burden is associated with certain Asian countries, where high population densities correlate with elevated air pollution levels [4]. Elevated levels of PM_2.5_, resulting from rapid economic growth and urbanization in past decades [5], are common in many regions of China [6,7,8,9,10], posing an unprecedented threat to regional air quality and human health [11,12].

In the past decades, a number of studies have addressed various aspects of particulate matter pollution in China, such as spatiotemporal variations [8,13], formation and evolution mechanisms [14,15,16], source apportionment [17,18], health effect assessments [12,19], and mitigation measures and policies [11,20]. Several nationwide studies have presented national maps of PM_2.5_ and key components measured at regional background locations or during field campaigns in major cities [8,10,21]. However, at the time this study was conducted, similar studies focused mostly on a few economically developed regions in China, including the Beijing–Tianjin–Tanggu area, the Yangtze River Delta, and the Pearl River Delta. Knowledge on characteristics and sources of PM_2.5_ pollution in other regions (e.g., northeastern China) are still limited, making it difficult to design effective emission control policies at local, regional, and even national levels.

This study presents detailed observations and analyses of PM_2.5_ and its chemical components during wintertime in Changchun, a metropolitan area located in the center of China’s breadbasket region. Wintertime air pollution in Changchun is shaped by several environmental settings unique to this region, including low temperatures, a shallow boundary layer, dense industrial emission sources, coal-dominant energy consumption, domestic heating with mixed fuel types (i.e., coal, wood), and frequent use of deicing salts [22]. Changchun is located in the middle of the Chinese “black soil” zone, one of the world’s three most fertile croplands. Temperatures in Changchun can be as low as −30 °C in winter, resulting in a planetary boundary layer so shallow that a virtual cap is formed. This phenomenon prevents dispersion of PM_2.5_ emitted from an increasing vehicle fleet and many industrial sources. Unlike heating in Beijing, domestic heating in Changchun is provided by both large and small coal- and wood-burning boilers, the emissions from which are more difficult to control than centralized heating systems. Changchun was one of the largest industrial hubs in China in the 1950s and 1960s, and many facilities are still operating with updated technology. For example, the car-making industry produces more than 2 million vehicles per year in this city. It is of interest to investigate how wintertime air pollution in Changchun is controlled by these environmental settings. To date, a number of similar studies conducted in northeastern China [23,24,25,26,27,28] have shown that PM pollution is very severe during winter. However, very limited studies on PM_2.5_ mass concentration [29] and its detailed chemical composition [8,30,31] in Changchun have been conducted so far.

In this paper, we present the detailed observations, taken during an intensive field campaign, of wintertime PM_2.5_ and its major components at four sites representing traffic, residential, campus and suburban village, respectively, in the Changchun metropolitan area. The objectives of the present study are three-fold: (1) to determine the spatial variability of PM_2.5_ levels in Changchun; (2) to investigate the chemical characteristics of PM_2.5_; and (3) to probe the main sources of PM_2.5_ during the study period. This study will provide useful information regarding PM_2.5_ pollution and its contributing sources in this relatively under-studied area. This information can be used to inform future decision making on mitigating particulate matter pollutions. The PM_2.5_ composition observations presented here are also expected to improve emission inventories of PM_2.5_ and provide reliable constraints for chemical transport models to predict PM_2.5_ pollution in Northeast China.

## 2. Materials and Methods

### 2.1. Description of the Sampling Sites

Changchun (124°18′ E–127°02′ E, 43°05′ N–45°15′ N) is located in the center of the Northeast Plain in China. The Changchun metropolitan area is comprised of 119 townships in 10 counties, with a total land area of 20,660 km^2^ and a population of 7.6 million, of which 3.6 million lived in urban areas in 2011 [22]. It is within the North Temperate Zone, which is characterized by a continental monsoon climate with cold, dry winters and warm, humid summers. The annual average temperature is 5.2 °C, and the average annual rainfall is 560 mm. The prevailing wind direction is southwest throughout the year, with a mean wind speed of 4.3 m/s. Temperature inversion, a meteorological condition conducive to degrading air quality when combined with intense local emissions, occurs frequently in winter [32].

In China, coal combustion has been identified as one of the largest contributors to air pollution [33]. In Changchun in 2011, a total of 25.8 million tons of raw coal were consumed, among which 13.8 million tons were consumed by thermal power generation and another 4.8 million tons were consumed by wintertime domestic heating [22]. The major industries in Changchun include the automotive industry, coal-fired power plants, metallurgy, iron and steel mills, machine manufacturing, and electric and electronics manufacturing, and most of these industries are located in the western and northern portions of Changchun [34].

**Figure 1:**
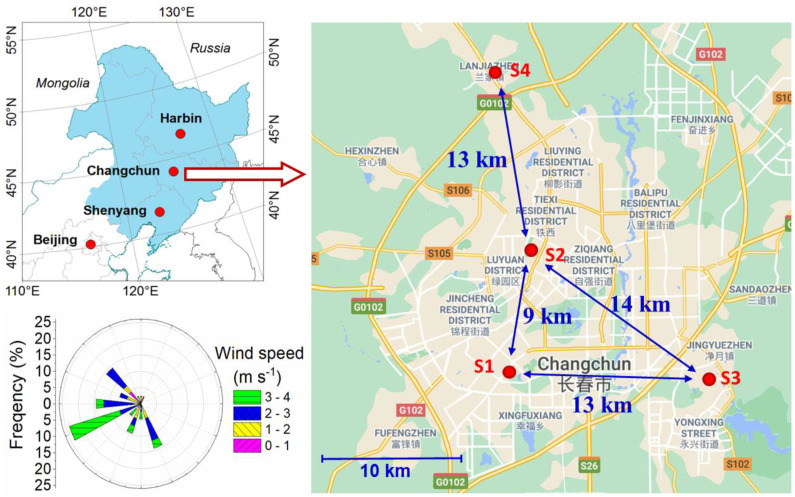
Schematic map of the study sites in Changchun city, Jilin Province, China. Detail information on meteorology and possible emission sources of the sites seen at Table 1. Blue color shaded area in the left upper panel indicates the area of Northeastern China, and the left lower panel is the wind rose map during the study period. The distances between the sites are also indicated.

**Figure 2:**
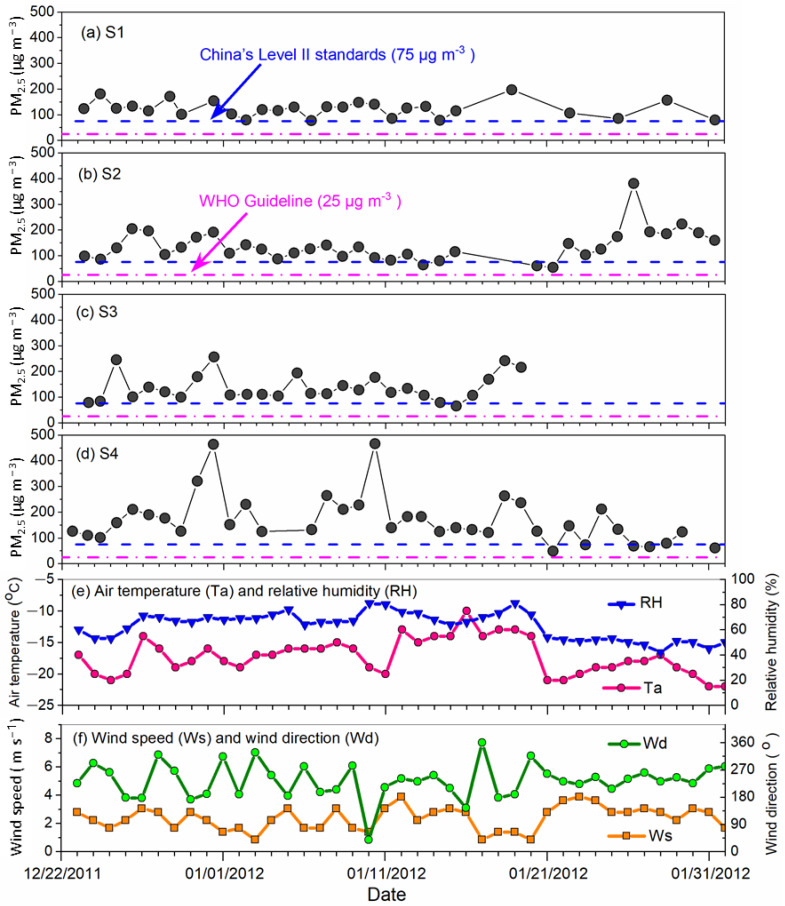
Temporal variation of PM_2.5_ mass concentration of the filter samples at sites S1 (**a**), S2 (**b**), S3 (**c**), S4 (**d**), air temperature and relative humidity (**e**), and wind speed and wind direction (**f**) during the sampling period.

A field campaign was carried out from 23 December 2011 to 31 January 2012. Ambient air was sampled at four sites in Changchun (Figure 1). These sites included the following locations: (1) the Chinese Academy of Sciences Institute of Geography and Agroecology (traffic site, marked as S1) located in the southern part of the Changchun industrial zone; (2) the Liaoyang Residential Area in the city center (residential site, S2); (3) the Jilin Agricultural University (campus site, S3) site at the southeastern corner of Changchun; and (4) the Guangning Village site (suburban village, S4), a suburban community north of Changchun. The locations of these sites are depicted in Figure 1, and detailed descriptions and the meteorological variables of these sites are provided in Table 1 and Figure 2, respectively.

### 2.2. Filter Sample Collection and Chemical Composition Analysis

Daily 24-h PM_2.5_ samples were collected at the four sampling sites on 47 mm Whatman Teflon filters using BGI samplers (Model Omni, BGI Inc., Waltham, MA, USA) at a flow rate of 5 L/min at about 9 AM local time. The filters were equilibrated in a drying apparatus (temperature 20–25 °C; RH 35–45%) for 48 h and then weighed with an electronic balance (Model XS105DU, Mettler Toledo Inc., Greifensee, Switzerland, reading precision 10 µg) before and after sampling. Sampling time, air temperature and air pressure were automatically recorded, and the standard sampling air volumes (V_std_) were calculated from these parameters recorded by the air samplers. The PM_2.5_ mass concentrations were calculated using the PM_2.5_ mass divided by V_std_. Samples were stored below 4 °C in a refrigerator until analysis to minimize evaporation of volatile components.

A total of 129 filter samples were collected and weighed for bulk PM_2.5_ mass over the whole campaign. However, 19 of the samples were accidentally lost before the laboratory analysis. Consequently, the remaining 110 samples were analyzed for chemical compositions. For data quality assessment and control (QA/QC), parallel sampling, a blank test, and a standard curve were performed during the outdoor sampling and laboratory analysis. For one half of each Teflon filter sample and field blank filter was extracted with 10 mL ultrapure water in an ultrasonic bath for 30 min. After passing through microporous membranes (pore size 0.45 mm, diameter 25 mm, Auto Science (Tianjin) Instrument Co., LTD, Tianjin, China), the extracts were stored at 4 °C in a precleaned tube for analysis. Four anions (F^−^, Cl^−^, NO_3_^−^ and SO_4_^2−^) and 5 cations (Na^+^, NH_4_^+^, K^+^, Mg^2+^ and Ca^2+^) were analyzed by Ion chromatography (ICS-1000, Dionex Inc., Sunnyvale, CA, USA), which consists of a separation column (Dionex Ionpac AS14 for anion and CS12A for cation) and a guard column (Dionex Ionpac AG14 for anion and AG12A for cation). A weak base eluent (3.5 mmol Na_2_CO_3_/1.0 mmol NaHCO_3_ solution) was used for anion detection, and 20 mmol methane sulfonic acid (MSA) solution was used for cation detection. Blank values were subtracted from samples. The recovery rates of the measured ions were in the range of 80–120%. The limits of detection are in Table S1, and uncertainties are ±10% for all ions.

Another half of each Teflon filter sample was dissolved at 170 °C for 4 h in a high-pressure Teflon digestion tank with mixed acid composed of 3 mL of concentrated HNO_3_, 1 mL of concentrated HClO_4_, and 1 mL of concentrated HF. After the solutions were cooled, 0.1 mL of concentrated HNO_3_ and deionized water were added to dilute the digestion liquid to 10 mL. A total of 18 elements (Al, Si, Ca, Fe, Mg, K, Mn, Ni, Cu, Zn, As, Se, Sr, Ba, Cd, Cr, Nd, and Pb) were measured using an inductively coupled plasma-atomic emission spectrometer (ICPE9000, Shimadzu Co. Ltd., Kyoto, Japan). The sample solutions were measured in triplicate, and standard and blank samples were inserted and analyzed every 10 samples for quality control.

### 2.3. Meteorological Data

Meteorological data, including ambient temperature (*T*_a_), pressure (*P*_a_), and relative humidity (*RH*), were measured using an automatic meteorological station (Model AMS-II-N, Changchun Meteorological Instrument Research Institute, Changchun, China) at the IGA site. Wind speed (*W*_s_) and wind direction (*W*_d_) were downloaded from a weather data archive (http://www.wunderground.com/history; accessed on 16 April 2021).

### 2.4. Coefficient of Divergence (COD) Analysis

In this study, spatial variability of PM_2.5_ among the sampling sites were tested through coefficient of divergence (COD) analysis:

(1) $$COD_{jk}=\sqrt{\frac{1}{p}\overset{p}{\underset{i=1}{\sum }}{\left(\frac{x_{ij}-x_{ik}}{x_{ij}+x_{ik}}\right)}^{2}}$$

where *x_ij_* and *x_ik_* represent the 24-h average PM_2.5_ concentration for day *i* at site *j* and site *k*, and *p* is the number of observations. A value of 0 for the *COD* implies that two sites are exactly alike, and a value larger than 0 implies that the two sites are dissimilar. Criteria of *COD* > 0.2 indicating heterogeneity and <0.1 indicating homogeneity were selected for the spatial variability analysis of PM_2.5_ [10,35].

### 2.5. Enrichment Factor (EF) Analysis for Elements

Here, the enrichment factor (EF) of the chemical elements was calculated to estimate the relative contributions from natural and anthropogenic sources [10,36,37].

EF = (X/X_ref_)_aerosol_/(X/X_ref_)_crust_ (2)

where (X/Al)_aerosol_ is the ratio of measured concentration of the element of interest (X) to aluminum (Al) in PM_2.5_; (X/Al)_crust_ is the ratio of X to Al in crustal material. The information of (X/Al)_crust_ was based on [38].

If the EF values of elements of interest are below 10, their sources are identified as predominantly natural sources; if the EF values are between 10 and 100, their sources are believed to be both natural and anthropogenic origins; and if the EF values are larger than 100, the dominance of anthropogenic are confirmed [36].

## 3. Results and Discussion

### 3.1. Levels of PM_2.5_ Concentrations in Wintertime Changchun

The daily average PM_2.5_ mass concentrations were 122.7 ± 30.9 µg m^−3^, 136.7 ± 60 µg m^−3^, 137.2 ± 50.1 µg m^−3^ and 170.6 ± 93.1 µg m^−3^ at sites S1, S2, S3 and S4, respectively, with an arithmetic average of 143.5 ± 68.1 µg m^−3^ for all sites (Figure 2 and Table 2). At all sites, the PM_2.5_ levels consistently exceeded the World Health Organization (WHO) air quality guidelines for PM_2.5_ (25 µg m^−3^) and China’s Level I and II air quality standards for PM_2.5_ (35 and 75 µg m^−3^, respectively). Our results show that although Changchun is not known as one of the most polluted cities in China, PM_2.5_ pollution in Changchun is comparable to PM_2.5_ pollution levels in severely polluted Chinese cities such as Beijing [10], Shanghai [39] and Guangzhou [9], as well as many other polluted Asian cities (Table 3).

As shown in Figure 2 and Table 2, large spatial variabilities in PM_2.5_ concentrations existed among the sampling sites. Coefficient of divergence (COD) analysis showed that the COD values of the site-pairs among the S1, S2, and S3 sites were lower than 0.2, indicating the spatial variabilities among these sites were small. However, the COD values between the S4 site and other three sites were all larger than 0.2 (Table S2), indicating the S4 site was a distinct site among the four sites.

Among the four sampling sites, the suburban (S4) site exhibited considerably higher PM_2.5_ concentrations than other sites (Figure 2 and Table 2). The higher PM_2.5_ level at the S4 site was likely due to the lack of effective emission control measures for local emission sources, e.g., coal and biomass burning for household heating and cooking, fugitive dust from unpaved surfaces, and processes at nearby individual workshops.

### 3.2. Temporal Variations of PM_2.5_ Concentrations

During the study period, heavy haze events with PM_2.5_ levels higher than 150 µg m^−3^ frequently occurred, as observed on Dec 30 and 31 in 2011 at all the four sites, 26 January 2012 at S2 site, 25 June 2012 at S3 and S4 sites, and 7, 10, 18, 19 January 2012 at S4 site (Figure 2). A few relatively clean days as observed at S2 and S4 sites were associated with the Chinese New Year (also known as “Spring Festival”), with the lowest PM_2.5_ concentration observed on 21 January 2012, which was Chinese New Year’s Eve.

**Figure 3:**
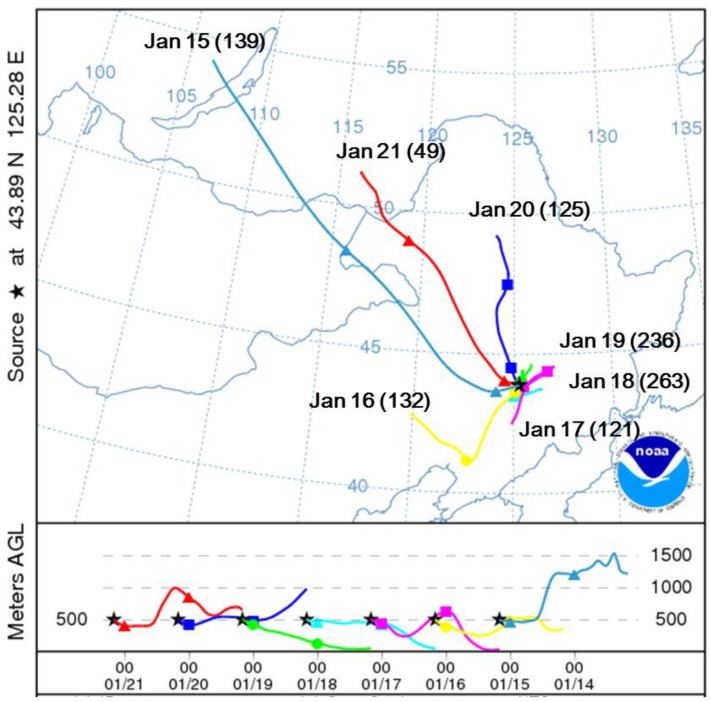
The evolution of 48-h backward trajectories commencing at 12:00 (local time) on 15 through 21 January 2012 at Changchun (43.89 N, 125.28 E) during which a haze event occurred on 18 and 19 January. The evolution of the trajectories is indicated by dates at the ends of the trajectories. The numbers in the brackets are PM_2.5_ mass concentrations at the S4 site for each day. The black lines in the lower panel are the terrain heights where the trajectories passed over.

On 18 and 19 January, a heavy haze occurred simultaneously at S1, S3 and S4 site with PM_2.5_ values nearly or higher than 200 µg m^−3^. Meteorological analysis (Figure 2) showed a sudden decrease of wind speeds from 2.8 m/s to 0.8 m/s on 17 January. Meanwhile, the 48-h back trajectory analysis using the HYSPLIT model [52,53] clearly shows that the air mass switched suddenly on 17 January from long range transport from southeast direction to local origins, as could be easily seen by the swift of Above Ground Level (AGL) of the air mass reaching the sampling site (Figure 3). From January 17 to 20, the static weather condition, coupled with relatively high air temperature and relative humidity, may have facilitated the accumulation of air pollutants and generation of secondary aerosols, resulting in the dramatic increasing of PM_2.5_ concentrations.

Over the whole campaign, correlation analysis (Figure S1) indicated that PM_2.5_ showed strong negative relationship with visibility, suggesting important influences of PM_2.5_ on haze pollution. Besides, PM_2.5_ showed an obvious negative correlation with wind speed owing to the dilution effects of wind on PM_2.5_ in the atmosphere. However, no obvious correlations were observed between PM_2.5_ and air temperature, relative humidity, dew point temperature and air pressure. Further analysis showed that the heavy hazy days were usually associated with winds from the directions of potential main emission sources as depicted in Table 1 (also see Figure S2).

**Figure 4:**
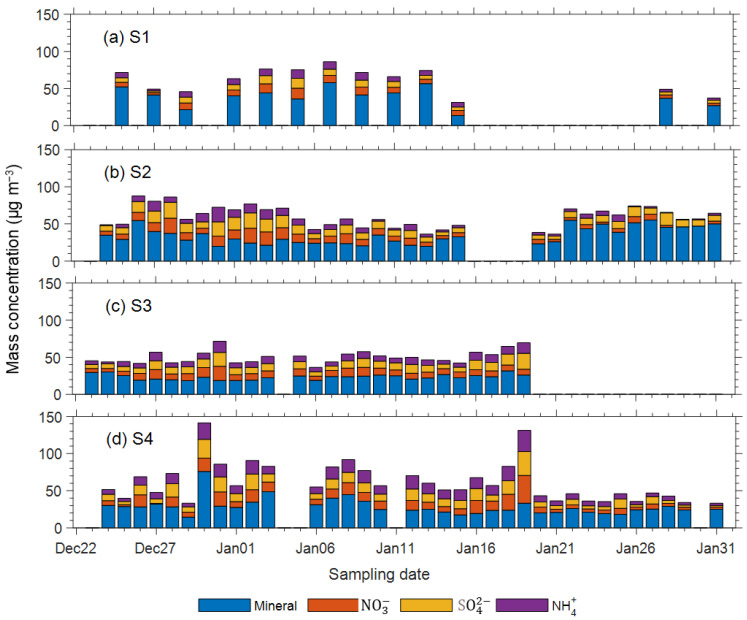
Temporal variations of mass concentrations of the mineral and secondary inorganic aerosols (SO_4_^2−^, NO_3_^−^, and NH_4_^+^) in the daily PM_2.5_ samples at sites S1 (**a**), S2 (**b**), S3 (**c**), and S4 (**d**).

**Figure 5:**
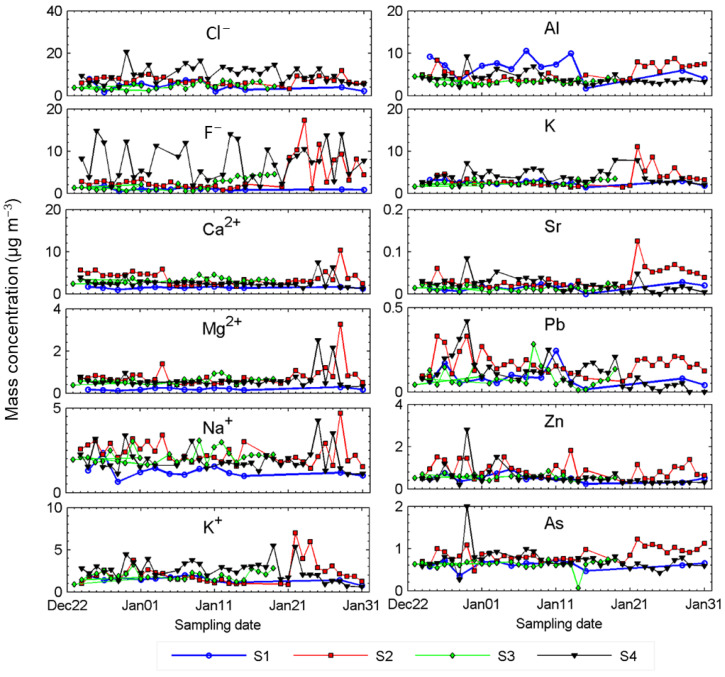
Temporal variations of mass concentrations of the representative ions and elements in the daily PM_2.5_ samples.

There is a distinctive temporal changes in PM_2.5_ at the residential (S2) site compared to other sites (Figure 2). The PM_2.5_ concentrations at the S1 and S4 sites decreased remarkably after 21 January, which was possibly related to the reduction of secondary inorganic aerosols (Figure 4d). However, the PM_2.5_ levels at site S2 increased noticeably during the same period, largely due to the increase of mineral dust (Figure 4b). As shown in Figure 5, the elements related to crustal (e.g., Al, K, and Sr), motor vehicles (e.g., Pb and Zn), coal combustion (e.g., As) and fireworks (e.g., Sr) also increased considerably after 21 January, indicating that human activities in the residential area during the Chinese Spring Festival celebration may have appreciably contributed to local PM_2.5_ pollution at the S2 site, regardless of the intrusion of cleaner background air.

### 3.3. Chemical Compositions of PM_2.5_

At all sites, mineral dust was the most abundant chemical component, contributing 13.6 µg/m^3^ to 75.9 µg/m^3^ to PM_2.5_ mass concentration, with an arithmetic average of 30.4 ± 11.4 µg/m^3^ (Table 2). Here the mineral dust in PM_2.5_ was calculated as the sum of Al, Si, Ca, Fe, Mg and K oxides (i.e., mineral dust = 1.89 Al +2.14 Si +1.40 Ca +1.43 Fe +1.66 Mg +1.21 K) [40], assuming that the elements existed as oxides [54]. In this study, the mineral levels were much comparable to those reported in other Chinese cities (e.g., 26.5 µg/m^3^ in Beijing [10]) and constituted the largest portion (20.7%) of total PM_2.5_ mass, indicating prevailing emission of soil dust particles in Changchun.

The secondary inorganic aerosols (SIA, including SO_4_^2−^, NO_3_^−^ and NH_4_^+^), with a mean of 27.6 ± 5.2 µg/m^3^, constituted the second largest portion (18.8%) of the total PM_2.5_ mass. This percentage was lower than some reported values in Beijing (28%) [8], Tianjin (34.1%) [36], and Guangzhou (48.5%) [55], but similar to or even higher than those in some other studies conducted in wintertime Beijing (12.0% in [10] and 20.5% in [55]), Harbin (13.7%) [56], and Changchun (18.7%) [31]. We suggest that the relatively low SIA/PM_2.5_ mass ratios in this study might indicate that the low temperatures in such cold regions have suppressed secondary inorganic formation in winter.

Another notable feature of the chemical characteristics of PM_2.5_ in the study period is high Cl^−^ (7.1 ± 3.5 µg/m^3^) and F^−^ (4.1 ± 4.0 µg/m^3^) loadings at all sites. To our knowledge, the observed F^−^ content (7.3 ± 4.2 µg/m^3^) at the suburban site (S4) was the highest value ever reported at an urban environment based on literatures. F^−^ concentration typically ranges from 0 to 2 µg/m^3^ [57,58]. Cl^−^ and F^−^ are usually emitted from coal combustion [57], while Cl^−^ can also be emitted from biomass burning in NE China [59]. Although coal burning is important in Changchun due to domestic heating, the unusually high levels of Cl^−^ and F^−^ concentrations could not be accounted for by coal burning alone because other co-emitted components [60] were not elevated proportionally. After examining the local emission sources, we speculate that the high levels of F^−^ observed in this study can be attributed to emissions from plastic-making and specialized glass-making facilities near the S4 site, where glass products were treated with an F solution for embroidery designs. Additionally, NH_4_^+^ at S4 was the highest among the four sites, likely due to its proximity to NH_3_ emission sources such as poultry farms.

Among the trace elements, As (0.7 ± 0.2 µg/m^3^), Zn (0.65 ± 0.4 µg/m^3^) and Ba (0.3 ± 0.2 µg/m^3^) were the most abundant in PM_2.5_. As is a marker for coal combustion and was present at much higher levels in this study than in other studies [61], suggesting that wintertime air pollution in Changchun may pose serious health threats to local residents given the well-known health effects associated with such trace elements. Zn and Ba are associated with non-tailpipe emissions, such as brake and tire wear [62]. Ba is also a good indicator of firework emissions [63,64]. The Zn concentrations observed in this study are comparable to those in polluted cities such as Shanghai [65] and the Beijing–Tianjin–Shijiazhuang city clusters [66] in China. Whereas the observed Ba concentration is much higher than those reported in the Beijing–Tianjin–Shijiazhuang city clusters [36,66], indicating contribution from fireworks emissions.

### 3.4. Temporal Variations of PM_2.5_ Components

For the S4 site, high SIA loadings on 18 and 19 January, and a sudden falling of SIA on 21 January were observed (Figure 4). We attributed such changes to both regional-scale transport and secondary formation processes from local precursors. From 17 to 19 January, the dominating air masses were from local origins (Figure 3) which provided a meteorological condition (low wind speed and high ambient temperature and humidity) that was favorable to growth and accumulation of SIA [67]. After three days of accumulation, SIA reached the maximum value on 19 January. Meanwhile, on 21 January, the dominating air mass origins switched to remote regions as far as Russia. Such air masses provided clean background air and a meteorological condition in favor of diluting the pollutants, resulting in a sudden decrease of SIA and total PM_2.5_ mass.

After 21 January, the mineral components increased notably at the residential (S2) site but decreased at other sites (Figure 5). The opposing temporal trends of mineral contents between S2 and other sites resulted in differences in PM_2.5_ trends between these sites, as mentioned above (Figure 2). The increase in crustal components at site S2 was unlikely to have been caused by windblown dust emissions from the strong wind during this period or by long-range transport of dust plumes from northern arid lands. During the campaign period, the surface in northern China was largely covered by snow and ice, and soil moisture was too high to allow dust mobilization. Long-range transport was also excluded because only the S2 site observed increased crustal element concentrations, whereas other sites that were likely influenced by dust plumes did not experience increases in crustal elements. Emissions from local sources, including fireworks and bridge and subway construction, resumed after the holidays in urban areas. In addition, fugitive dust was re-suspended by human activities under windy conditions. These factors were the major reasons for the increase in crustal PM_2.5_ concentrations at the S2 site.

Figure 5 illustrates the temporal variations of the measured ions other than SIA and crustal elements. In general, these elements showed larger variations at the S2 and S4 sites than at the other locations, which is consistent with the variable trends in PM_2.5_ concentrations. Site S2 exhibited high loadings of mineral elements (e.g., Al, K), coal ash indicators (e.g., As) and firework indicators (e.g., Sr) after 21 January, which is consistent with the aforementioned temporal trends of mineral PM_2.5_ (Figure 4) and overall PM_2.5_ (Figure 2). The S4 site exhibited extremely high values for almost all elements on 30 December, the day with a very high PM_2.5_ value (321 µg m^−3^). However, high ion and element loadings were not detected on the next day (31 December) when the PM_2.5_ concentration peaked (reaching 463 µg m^−3^), suggesting that the peak was caused by different emission sources than those on the previous day. The abrupt changes in PM_2.5_ concentrations and chemical compositions highlighted the complexity associated with understanding the sources and behaviors of aerosol pollution in this area.

### 3.5. Source Characteristics of PM_2.5_

As shown in the above sections (Table 2; Figure 2), in conjunction with COD analysis, large spatial variabilities in PM_2.5_ concentrations were observed among different locations, suggesting different source origins of PM_2.5_ at different locations. Among the sampling sites, the S2 site and especially the S4 site showed obvious distinction. The large spatial variations in PM_2.5_ might come from a couple of causes: (1) The samples over the campaign were sparse, resulting in limited number of pairs ranging from 24 to 33 and thus larger COD values; (2) The sampling sites were far from each other (9–14 km) and characterized with significantly different surroundings, which increased spatial variabilities of PM_2.5_; (3) Contributions from primary emission sources, i.e., mineral dusts (20.7%), were relatively more important than those from secondary inorganic aerosols, i.e., SIA (18.8%), causing sensitive responses of PM_2.5_ to primary sources and thus relatively large spatial variabilities. Indeed, four sampling sites were far less than needed to fully represent the spatial variabilities of PM pollution in such a metropolitan region with a fully urbanized area larger than 1200 km^2^ (https://en.wikipedia.org/wiki/Changchun; accessed on 16 April 2021). Actually, nine sampling stations in urban area and one station in outer suburb have been in running by Bureau of Ecology and Environment of Changchun since 2013.

**Figure 6:**
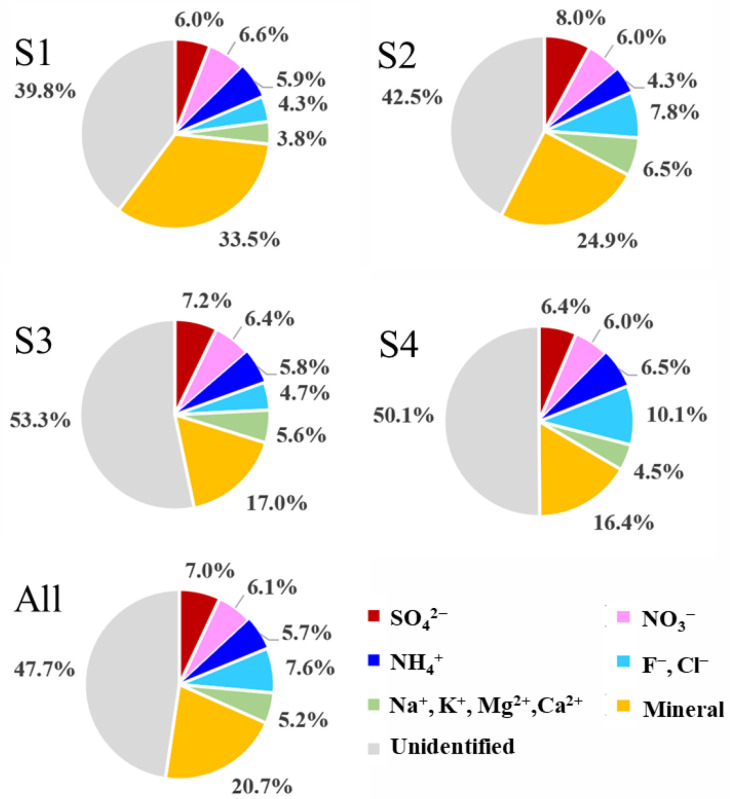
Pie-chart showing the chemical compositions (in %) of PM_2.5_ mass concentrations for the sampling sites.

From the results of chemical analysis (Table 2; Figure 4 and Figure 5), and the constructed mass closure of PM_2.5_ (Figure 6), the PM_2.5_ sources of the sampling sites also showed obvious spatial variabilities. For example, the S1 site showed highest mineral components and NO_3_^−^/SO_4_^2−^ mass ratio, consistent with its source features as influenced significantly by traffic; the S2 site showed high SO_4_^2−^, As and mineral, indicating the prominent contributions from coal burning and traffic; the S4 site was characterized by high halogen species, K^+^ and NH_4_^+^, indicating the contributions from multi-sources such as coal burning, glass sculpture, biomass burning, and agricultural sectors.

**Figure 7:**
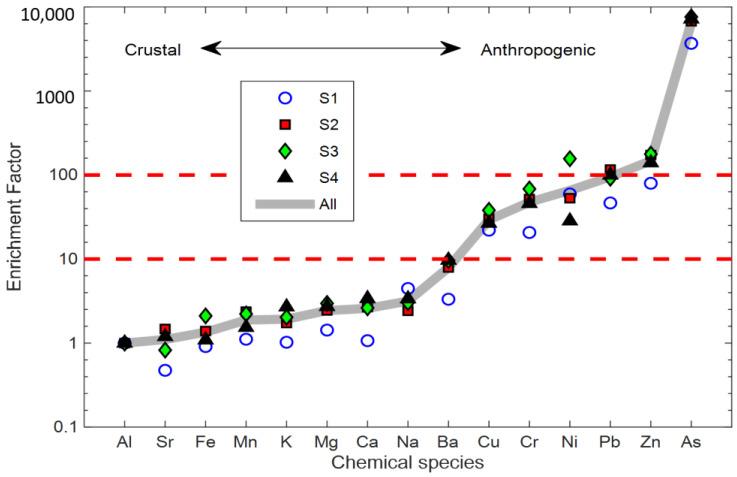
Enrichment factors calculated using Al as a reference element during the whole sampling period.

The enrichment factor (EF) analysis for the elements was also used to estimate the relative contributions of natural and anthropogenic origins to wintertime PM_2.5_ in Changchun, following the methods developed by [38]. The results (Figure 7) show that the calculated EF values of Al, Si, Fe, Mn, K, Mg, Ca, and Na were all below 10, suggesting that these elements came predominantly from natural sources. The EF values of Ba, Cu, Cr, Ni, and Pb were between 10 and 100, suggesting contributions from both natural and anthropogenic sources. The EF values of Zn and As were larger than 100, indicating the dominance of anthropogenic sources such as traffic, coal burning, and industrial processes.

In summary, the PM_2.5_ sources in Changchun were characterized with high spatial variabilities among different sites and noteworthy contributions from mineral and halogen species (Figure 6). In addition, the EF analysis showed high EF values of the As element. The chemical and source characteristics of PM_2.5_ were consistent with Changchun as being a typical coal burning and industry city.

### 3.6. Implications for the Effectiveness of Haze Pollution Control Measures

**Figure 8:**
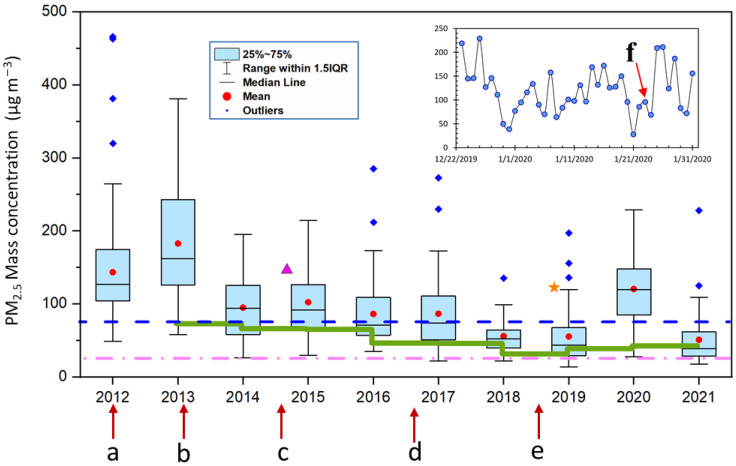
Long-term variations of PM_2.5_ concentrations in periods from 23 December to 31 January next year (box plots), year-round averages of PM_2.5_ concentrations (solid green line), and other published PM_2.5_ concentrations from Fang et al., 2017 (pink triangle) and Bai et al., 2020 (orange star). Letters below the x axis indicate related events: a. The observation campaign was conducted from 23 December 2011 to 31 January 2012 (this study); b. Implementation of the new released China’s Air Quality Standard in 2013, which included PM_2.5_ as a new criterion pollutant for the first time; c. Action Plan of Air Pollution Prevention and Control in 2014.6; d. Clean Air Action Plan in 2016.7; e. Three-Year Blue Sky War in 2018.9; f. The day (23 January 2020) when Wuhan, China released announcement of lock-down due to the COVID-19 epidemic.

To estimate the effectiveness of haze mitigation measures dating back to 2012, historic air quality data in Changchun from Chinese Environmental Protect Agency were collected (https://www.aqistudy.cn/historydata/; accessed on 16 April 2021). Results in Figure 8 show that wintertime PM_2.5_ pollution was very severe in 2012 and 2013, and decreased significantly in 2014 after implementing the new Chinese National Ambient Air Quality Standard (NAAQS) in 2013, but still stayed above Level II (75 μgm^−3^) of NAAQS. There was an obvious decreasing trend in both annual averages and wintertime level of PM_2.5_ from 2013 to 2018, synchronizing well with the implementation of major air quality control and prevention measures, i.e., implementation of NAAQS in 2013, Action Plan of Air Pollution Prevention and Control in 2014, Clean Air Action Plan in 2016 and Three-Year Blue Sky War in 2018. Such synchronization probably indicated that the measures taken by local government had exerted remarkable effects in cutting down the fine particle pollution in the Changchun area.

However, the PM_2.5_ switched to a slightly increasing trend from 2019. Even more, the wintertime PM_2.5_ in the period between 23 December 2019 and 31 January 2020 reached the highest level since 2014. The PM_2.5_ level appears to reach a stationary plateau stage, with PM_2.5_ loadings ranging between 51 and 56, and greater efforts must be made to further lower the pollution level in the future.

## 4. Conclusions

This study presents detailed field observations of the chemical components and spatio-temporal variations of wintertime PM_2.5_ in the Changchun metropolitan area located in the center of the China’s breadbasket region. Such information could be used as a baseline for related studies based on the fact that such information for this region was very limited before 2013. We found that severe wintertime haze pollution existed in Changchun due to unique environmental settings, including strong temperature inversion, high emission loadings, scattered sources with mixed fuel types, and frequent use of deicing salts. Observations from the four sites across the city revealed that PM_2.5_ levels (143 µg m^−3^) constantly exceeded the WHO air quality guidelines for PM_2.5_ (25 µg m^−3^) and China’s Level I (35 µg m^−3^) and II (75 µg m^−3^) air quality standards for PM_2.5_, with levels comparable to those observed in Asia’s most polluted cities. Mineral dust and secondary inorganic aerosols together contributed about 40% to the total PM_2.5_ mass.

While strong dependence of PM_2.5_ levels on atmospheric circulation were found, the observed heterogeneous distribution of PM_2.5_ mass and chemical compositions among the monitoring sites suggested that localized emission sources significantly affected PM_2.5_ pollution in Changchun. In particular, the suburban village site exhibited the highest PM_2.5_ levels and extremely high Cl^−^ and F^−^ loadings.

Results from this study shed light on both the severity of air pollution in China and the potential measures required to understand and mitigate air pollution in this region. The fact that the levels of both total PM_2.5_ and its inorganic components were comparable to those in China’s most polluted cities was surprising because Changchun has generally been considered less polluted than other large Chinese cities. In fact, the sky was blue and sunlight was dominant during the field campaign, even on the most polluted days, unlike the visible haze in other cities. However, pollution was visible from a horizontal viewpoint. Our results suggest that there is likely a mechanism behind the high pollution episodes in this region that is distinct from the mechanisms in the Beijing–Tianjing–Hebei region or the Pearl River Delta region. The distinct characteristics of air pollution in this region also make pollution difficult to detect using satellite remote sensing or to simulate using numerical chemical transport models. The shallow boundary layer confines most of the aerosols to the lowest atmospheric layer, and the land in this region is largely covered by snow and ice during the cold season, making it challenging to remotely sense atmospheric aerosols. Furthermore, emission inventories in China have focused on typical industrial sources derived from province-level [68] or county-level [69] energy consumption. Chemical transport models based on these inventories are likely to under-predict some major components of PM_2.5_ unless the missing sources can be accurately accounted for in the emission inventories used to drive these models.

Based on the results of the chemical component analysis, one notable characteristic of air pollution in wintertime Changchun was high loadings of mineral dust, other than the chemical species related to the burning of fossil fuels. Thus, effective measures, i.e., more thorough and frequent sweeping or watering of streets, covering of bare soil on sides of busy roads, and stricter dust controls for the widespread construction activities, should be taken to control the road and off-road dust emissions. In addition, the comparisons among the monitoring sites showed that the upwind suburban sites were more polluted than the sites in the urban area. In the future, more attention should be paid to the emissions from small factories of the township enterprises in the suburban and even rural areas.

## Figures and Tables

**Table 1:** Description of the sampling sites in this study.

Site	Type	Geographic Location	Description
S1	Traffic	43°48′38.33″ N, 125°14′51.20″ E	Southwest of Changchun city, about 30 m north of the 3rd South Ring Road, and 250 m east of the Guigu Street of Changchun city. 24-h aerosol samples were collected using a PM_2.5_ sampler, at a height of 3.0 m above the ground in Northeast Institute of Geography and Agroecology, Chinese Academy of Sciences. No main PM sources other than vehicle and road dust were presented at the site.
S2	Residential	43°53′21.51″ N, 125°16′36.33″ E	Northwest of Changchun city, about 200 m west of the 2nd West Ring Road of Changchun city. The 24-h aerosol samples were collected on an 8-m tall building in the Liaoyang residential community. Lots of residential buildings and small to medium sizes of stores and restaurants along the streets nearby. A coal-fired boiler for heating located about 100 m west of the sampling site. The sources of PM were a mixture from road and construction dust, coal burning, tailpipe emissions and food cooking.
S3	Campus	43°48′28.29″ N, 125°24′53.09″ E	Southeast of Changchun city. 24-h aerosol samples were collected using a PM_2.5_ sampler, at a height of 3.0 m above the ground within the yard of an edible fungi research and teaching in campus of Jilin Agricultural University, about 200 m west of the Feihong Road. Fugitive dust and activities related to edible fungi planting and research may be the main emission sources of PM.
S4	Suburban village	44°0′41.61″ N, 125°15′14.02″ E	Northwest of Changchun city. 24-h aerosol samples were collected using a PM_2.5_ sampler, at a height of 3.0 m above the ground in the yard of a farmer’s family in a village of Lanjia town. The sampler was surrounded by some 5-m high buildings about 10 m away. About 500 m away from the main road of Lanjia Town with many small factories. Road dust, biomass burning, cooking, and emissions from the nearby factories in Lanjia Town were the main sources of air pollutants.

**Table 2:** Average mass concentrations (mean ± standard deviation) of PM_2.5_ and corresponding chemical compositions at four sites.

Chemical Species	S1	S2	S3	S4	Average
			(µg m^−3^)		
PM_2.5_	122.7 ± 30.9 ^c^ (76.2–197.0)	136.7 ± 60.0 (54.4–381.4)	137.2 ± 51.7 (65.8–255.6)	170.6 ± 93.1 (48.7–466.0)	143.5 ± 68.1 (48.7–466.0)
NH_4_^+^	7.0 ± 2.9 (2.4–11.5)	6.0 ± 4.3 (0.8–19.8)	8.0 ± 2.9 (2.5–15)	11.3 ± 6.0 (3.2–28.6)	8.3 ± 5.0 (0.8–28.6)
Ca^2+^	1.5 ± 0.2 (1–1.7)	3.8 ± 1.7 (1.7–10.4)	3.1 ± 0.6 (2.2–4.6)	2.6 ± 1.2 (1.3–7.4)	3.0 ± 1.4 (1–10.4)
K^+^	1.5 ± 0.4 (0.8–2)	2.1 ± 1.3 (0.9–7)	1.9 ± 0.5 (0.9–3.3)	2.6 ± 1.2 (0.6–5.5)	2.1 ± 1.1 (0.6–7)
Na^+^	1.3 ± 0.4 (0.6–2.3)	2.3 ± 0.7 (1.4–4.7)	2.1 ± 0.4 (1.6–3.1)	1.9 ± 0.7 (1.1–4.3)	2.0 ± 0.7 (0.6–4.7)
Mg^2+^	0.2 ± 0.1 (0.1–0.3)	0.8 ± 0.5 (0.4–3.3)	0.6 ± 0.1 (0.4–1)	0.6 ± 0.4 (0.3–2.5)	0.6 ± 0.4 (0.1–3.3)
NO_3_^−^	7.8 ± 3.5 (3–14.5)	8.3 ± 5.1 (0–20.3)	8.9 ± 2.9 (4.6–19.1)	10.4 ± 7.1 (1.1–37.5)	9.0 ± 5.3 (0–37.5)
SO_4_^2−^	7.1 ± 3.1 (2.2–13.4)	11 ± 4.5 (4.5–21.5)	10 ± 3.8 (5.4–21.4)	11.1 ± 6.7 (2.2–32.1)	10.3 ± 5.1 (2.2–32.1)
NO_3_^−^/SO_4_^2−^	1.11 ± 0.17 (0.77–1.49)	0.74 ± 0.28 (0–1.12)	0.94 ± 0.23 (0.37–1.32)	0.95 ± 0.26 (0.18–1.56)	0.9 ± 0.28 (0–1.56)
Cl^−^	4.6 ± 2.1 (1.7–7.9)	7.0 ± 1.8 (3.2–11.8)	4.5 ± 1.5 (2.3–7.1)	10.1 ± 3.8 (4.1–20.5)	7.1 ± 3.5 (1.7–20.5)
F^−^	1.0 ± 0.3 (0.5–1.8)	3.8 ± 3.7 (0.8–17.3)	2.0 ± 1.4 (0.5–4.5)	7.3 ± 4.2 (1.5–14.8)	4.1 ± 4.0 (0.5–17.3)
Mineral ^a^	39.5 ± 12.9 (13.6–57.8)	34.3 ± 11.5 (19.8–55.1)	23.4 ± 3.7 (18.6–31.7)	28.4 ± 11.1 (14.4–75.9)	30.4 ± 11.4 (13.6–75.9)
Trace ^b^	0.8 ± 0.2 (0.5–1)	1.3 ± 0.3 (0.7–2)	0.9 ± 0.1 (0.7–1.3)	1 ± 0.5 (0.4–3.4)	1.05 ± 0.4 (0.43–3.42)
*n*	28	36	28	37	129

^a^ The mineral dust in PM-_2.5_ was calculated as the sum of oxides of Al, Si, Ca, Fe, Mg, and K (i.e., Mineral dust = 1.89Al +2.14Si +1.40Ca +1.43Fe +1.66Mg +1.21K) [40], of which the concentration of Si was estimated according to the average ratio of Si/Al (3.6) in earth’s crust [40,41,42]; ^b^ Trace elements include Mn, Ni, Cu, Zn, As, Se, Sr, Ba, Cd, Cr, Nd, and Pb; ^c^ numbers in bold are the average values and standard deviations, and numbers in the brackets are the observed minimum and maximum values. Site codes seen at Figure 1.

**Table 3:** Comparison of PM_2.5_ and PM_10_ mass concentrations in Northeastern China with other regions.

City	Station Type	Year	Season/Period	PM_10_ (µg m^−3^)	PM_2.5_ (µg m^−3^)	Measuring Method/Instrument	Reference
**Northeastern China**							
Changchun	Urban	2012	Winter		132.6 ± 50.6	Teflon filter gravimetric method	This study
	Rural	2012	Winter		170.6 ± 93.6	Teflon filter gravimetric method	This study
	Urban	2003	Winter		140.5 ± 28.6	Quartz filter gravimetric method	[43]
	Urban	2014	Autumn		146.0 ± 107.0	Quartz filter gravimetric method	[31]
	Urban	2018	Winter		121.8 ± 101.7	APDA-375A	[29]
Baicheng	Semi-arid	2006	Spring		260.9 ± 274.4	Sartorius MC5 electronic microbalance	[23]
Harbin	Urban	2008–2009	Winter (haze episode)	308 ± 167		Quartz filter gravimetric method	[26]
	Urban	2006–2007	Winter	155.1		Quartz filter gravimetric method	[44]
	Rural	2006–2007	Whole year	81.8		Quartz filter gravimetric method	[45]
Shenyang	Urban	2010–2012	Winter	88.0 ± 23.5	69.5 ± 19.9	GRIMM180	[27]
	Urban	2004–2005	Winter		132	Teflon filter gravimetric method	[24]
**Other region of China**							
Beijing	Urban	2005–2007	Winter		91 (63–112)	TEOM ^a^	[46]
	Urban	2010	Winter		139 ± 86	Teflon filter gravimetric method	[10]
Shanghai	Urban	2009–2010	Winter		122	Glass filter gravimetric method	[39]
Guangzhou	Urban	2004	Autumn		153.9 ± 52.1	TEOM (1400)	[9]
Xi’an	Urban	2006	Winter		266.8	Quartz filter gravimetric method	[47]
**Other countries**							
Ulaanbaatar	Urban	2008	Winter		105.1 ± 34.9	Teflon filter gravimetric method	[48]
New Delhi	Urban	2011	Whole year		122.3 ± 90.7	C14 BETA (FH 62 C14)	[49]
Bangkok	Urban	2010	Winter (dry season)		55 (16–103)	Quartz filter gravimetric method	[50]
Reno	Urban	2008–2010	Winter		15 (2–18)	BAM ^b^	[51]

^a^ TEOM: Tapered Element Oscillating Microbalance; ^b^ BAM: beta attenuation monitor.

## Data Availability

The data presented in this study are within the article or supplementary materials.

## Supplementary Materials

The following are available online at https://www.mdpi.com/article/10.3390/ijerph18084354/s1, Figure S1: Correlation relationships between filter based PM_2.5_ and related meteorological factors, Figure S2: Contouring plot showing the relationship between measured PM2.5 and wind speed and direction for the four sampling sites., Table S1: Method detection limit (MDL) for measuring the concentrations of 9 ions and 18 elements, Table S2: Coefficients of Divergence (COD) and pearson correlation analysis for the bulk PM2.5 concentrations between site pairs.
