# Supplementary material for: Chemical Composition and Source Apportionment of Wintertime Airborne PM2.5 in Changchun, Northeastern China
Source: Int J Environ Res Public Health. 2021 Apr 20;18(8):4354. doi: 10.3390/ijerph18084354 (PMC8073655; doi:10.3390/ijerph18084354)
Supplement: Supplementary file 1 [file ijerph-18-04354-s001.pdf]

Supplementary Materials

Table S1 Method detection limit (MDL) for measuring the concentrations of 9 ions and 18 elements in this study.

Species	MDL ($\mu g\ mL^{-1}$)	Species	MDL ($\mu g\ mL^{-1}$)
Na^+	0.0163	Cr	0.0012
NH_4^+	0.0327	Cu	0.0024
K^+	0.0357	Fe	0.0006
Mg^{2+}	0.0193	K	0.0029
Ca^{2+}	0.0126	Mg	0.0004
F^-	0.0200	Mn	0.0001
Cl^-	0.0338	Na	0.0029
NO_3^-	0.0892	Nd	0.0031
SO_4^{2-}	0.1107	Ni	0.0020
Al	0.0075	Pb	0.0053
As	0.0447	Se	0.0170
Ba	0.0004	Sr	0.0019
Ca	0.0037	Zn	0.0010
Cd	0.0009	---	---

Table S2 Coefficients of Divergence (COD) and pearson correlation analysis for the bulk $PM_{2.5}$ concentrations between site pairs

**Variables**	**S1-S2**	**S1-S3**	**S1-S4**	**S2-S3**	**S2-S4**	**S3-S4**
**number of pairs**	27	24	26	24	33	26
**COD**	**0.190**	**0.167**	**0.257**	**0.175**	**0.302**	**0.232**
r	**-0.012**	**0.4217**	**0.348**	**0.360**	**-0.027**	**0.610**
***p*-value**	0.9529	0.0402	0.0815	0.0842	0.8469	0.0009

Note: number of samples were 28, 36, 28 and 37 at S1, S2, S3 and S4 sites, respectively.

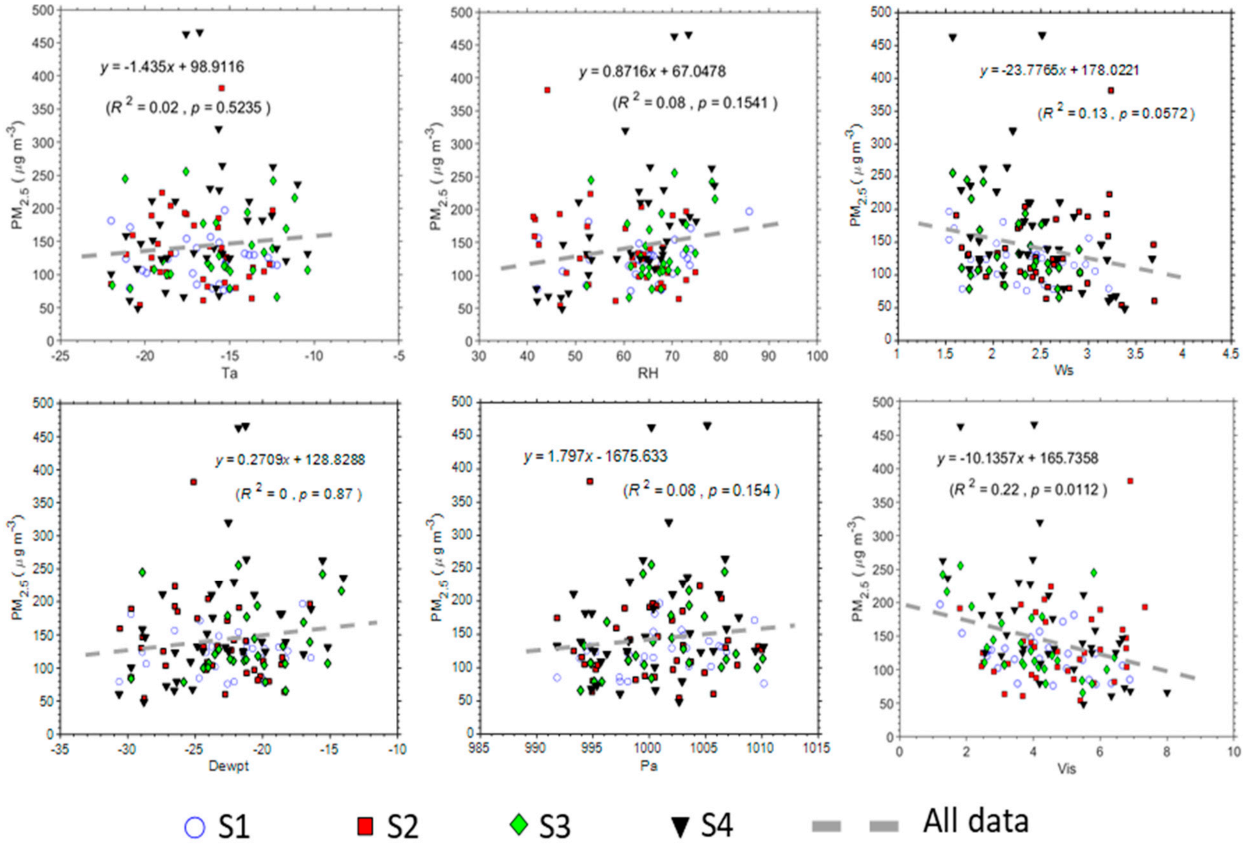

Figure S1. Correlation relationships between filter based $PM_{2.5}$ and related meteorological factors. Ta: air temperature (°C); RH: relative humidity (%); Ws: wind speed (m/s); Dewpt: dew point temperature (°C); Pa: air pressure (hPa); Vis: visibility (km).

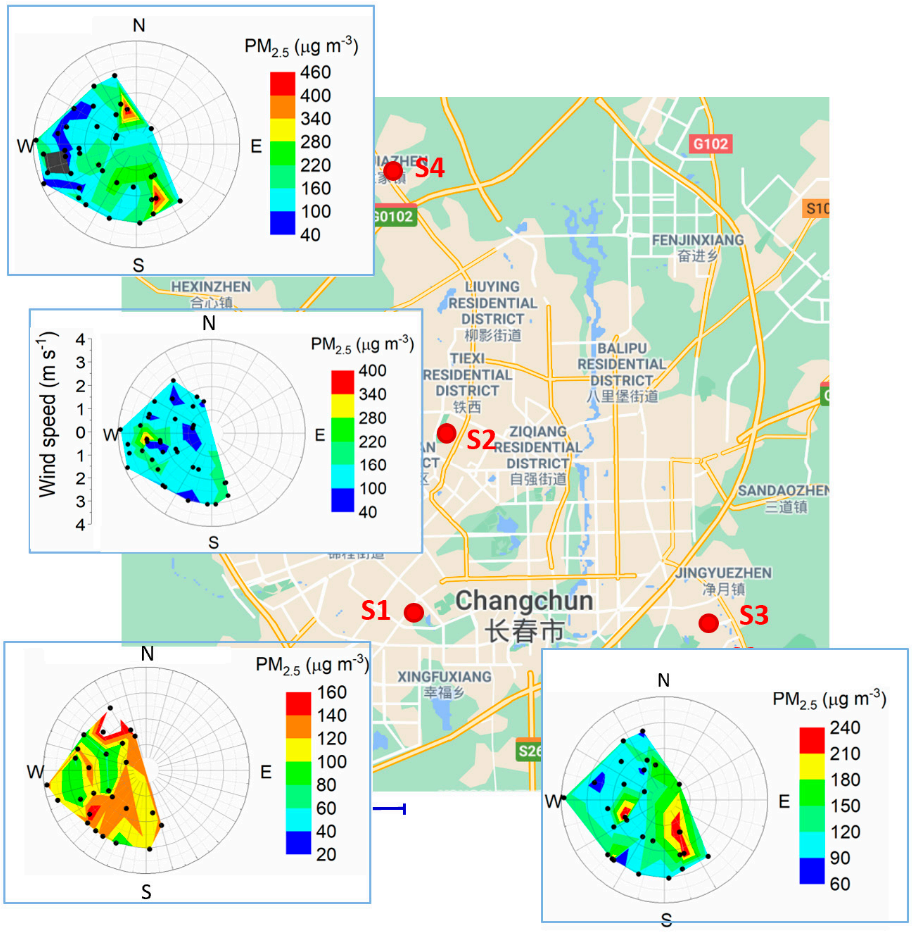

Figure S2. Contouring plot showing the relationship between measured $PM_{2.5}$ and wind speed and direction for the four sampling sites.
